# Gastric Neuroendocrine Tumor Presenting Atypically in von Hippel–Lindau Disease: A Case Report

**DOI:** 10.15586/jkc.v13i1.420

**Published:** 2026-03-31

**Authors:** Jiawei Chang, Qiong Zhang, Zhihui Liu, Yinmiao Bai, Hongchen Ji, Hong-Mei Zhang

**Affiliations:** Department of Oncology, the First Affiliated Hospital of AFMU of Airforce Medical University, Shaanxi, China

**Keywords:** germline mutation, HIF-alpha, neuroendocrine tumor, von Hippel–Lindau disease

## Abstract

von Hippel–Lindau (VHL) disease, an autosomal dominant inherited disorder resulting from mutations in the VHL gene, is known to be associated with the development of neuroendocrine tumors (NET) in various organs, including the adrenal gland, pancreas, and paraganglion. However, the development of gastric NET (gNET) in VHL disease has not been reported. Limited studies have suggested that clear cell change is a distinctive feature of VHL-associated tumors. Herein, we first report a case of a patient with VHL syndrome who presented with a gNET showing clear cell change, an unusual morphological characteristic of gNET, and harbored a pathogenic germline VHL mutation (c.351 G>T). The patient underwent surgical treatment for retinal hemangioblastoma, gNET, and renal cell carcinoma, in addition to receiving endocrine therapy and antiangiogenic drugs. The patient survived for 17 years. Our case highlights the possibility of VHL-associated NET development in uncommon locations. Further studies are required to elucidate the correlation between VHL mutation sites and the clinical manifestation of the disease.

## Introduction

von Hippel–Lindau (VHL) disease is an autosomal dominant neoplastic disorder characterized by the presence of a germline mutation in the VHL gene located on chromosome 3 (1, 2). The estimated incidence rate of VHL disease is approximately 1 in 36,000 (1). The syndrome mainly presents with cyst formation in the kidney and pancreas (3). Additionally, VHL disease is associated with the development of various tumors, particularly clear cell renal cell carcinoma (ccRCC), hemangioblastomas of the central nervous system and retina, and a range of neuroendocrine tumors (NETs), including pheochromocytomas, paragangliomas, and pancreatic NETs (pNETs) (4). Remarkably, approximately 60% of NETs identified in VHL disease exhibit a clear cell morphology with multivacuolated lipid-rich cell changes (5). However, the correlation between VHL disease and gastric NETs (gNETs) has not been extensively investigated. This case study presents a rare instance of a VHL-associated gNET, characterized by a germline mutation in the VHL gene.

## Case Report

The current case pertains to a 35-year-old Asian male who was admitted to our hospital in June 2006, presenting with symptoms of dizziness and pathobolism. The patient had no previous medical history. He reported that his mother had died from an “abdominal tumor,” although no pathological report was available to confirm this claim. The patient had a 17-year history of smoking three cigarettes per day but denied any history of alcohol or substance abuse. Magnetic resonance imaging (MRI) revealed a 5 × 4 cm tumor in the right cerebellum. The patient’s symptoms were alleviated following surgical resection of the cerebellar lesion. Histopathological examination identified the tumor as a hemangioblastoma. Two years later, the patient was diagnosed with type 2 diabetes and was prescribed subcutaneous insulin injections and oral acarbose for the management of blood glucose levels.

**Figure 1:**
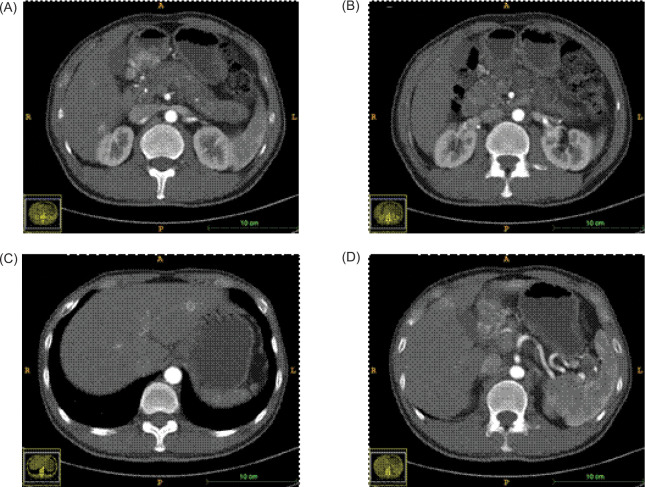
CT scanning during the first diagnosis (arterial phase). (A) lesion in the gastric antrum; (B) lesion in the left renal hilus; (C, D) lesions in the liver.

**Figure 2:**
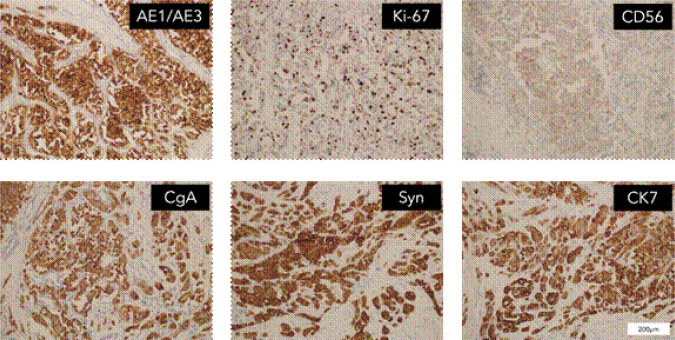
IHC staining for surgical specimens at the gastric antrum.

The patient demonstrated nonadherence to the regular follow-up appointments recommended by the healthcare provider. In July 2018, the patient reported experiencing abdominal pain. A contrast computed tomography (CT) scan disclosed a tumor in the antrum of the stomach, suggesting a neuroendocrine tumor, with associated metastatic tumors identified in the liver and the right renal area (Figure 1). Additionally, the CT scan revealed the presence of polycystic pancreas and polycystic kidneys. The patient underwent a gastroscopic biopsy of the gastric lesion, and immunohistochemical tests yielded positive results for Syn, CgA, and CD56, confirming the tumor as a neuroendocrine tumor. Furthermore, hyaline changes were observed in the tumor tissue (Figure 2). A subsequent 68Ga octreotide positron emission tomography (PET)-CT scan confirmed high metabolic activity in the tumors of the gastric antrum and liver, but not in the right renal area. Considering the patient’s previous diagnosis of cerebellar hemangioblastoma and these imaging findings, the probability of VHL disease was regarded as high. Genetic analysis using peripheral blood DNA identified a germline mutation in the VHL gene (c.351 G>T; p.Trp117Cys) and the POLE gene (c.C122T; p.Thr41Met). Genetic testing of the patient’s offspring revealed that his son carried the same mutations, while his sister did not display these mutations.

**Figure 3:**
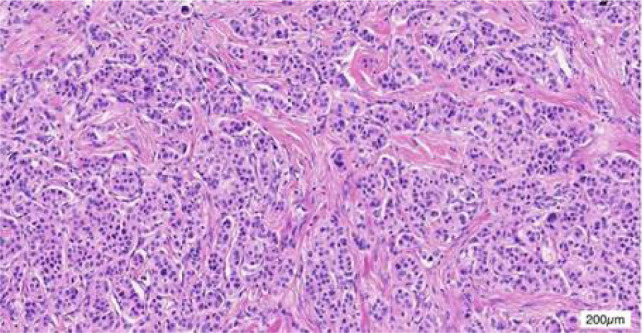
HE staining for surgical specimens at the gastric antrum.

In September 2018, the patient underwent a surgical resection procedure, entailing the removal of the distal stomach, a portion of the liver, and the gallbladder. The histopathological analysis of the surgical specimen affirmed the morphology of NET, with immunohistochemical staining for CgA, Syn, and CD56, thereby validating the NET diagnosis. The gastric lesion exhibited approximately 15% positive Ki-67 staining, indicating a G2 classification. Chemotherapy consisting of capecitabine and temozolomide was administered over five cycles from December 2018 to April 2019.

In June 2019, a robot-assisted resection was carried out for a 2 cm right renal tumor, which was diagnosed as ccRCC, in line with VHL disease. The patient began treatment with sunitinib (37.5 mg orally per day) from August 2020 to deal with the NET and ccRCC. However, the dosage of sunitinib was reduced to 25 mg per day due to elevated urine protein levels. In September 2021, liver metastases were identified during a follow-up CT examination, and 68Ga octreotide PET-CT revealed somatostatin receptor metabolic activity. A liver metastasis biopsy confirmed the existence of NET, and the lesion was positive for SSTR2 in immunohistochemical staining. Octreotide acetate microsphere injection treatment was initiated and lasted until December 2022, while sunitinib treatment was restarted in June 2022.

During the subsequent follow-up period, the patient displayed a continuous increase in the quantity and size of liver lesions from June 2021 to November 2022. A newly measurable lesion in lymph node metastasis was identified on November 20, along with the emergence of ascites and a diagnosis of secondary hypothyroidism. Subsequently, the patient refused any further anticancer treatment and was lost to follow-up in April 2023. The survival time since the diagnosis of cerebellar hemangioblastoma was 17 years, while that from the diagnosis of gNET and ccRCC was 5 years.

## Discussion

Gastroenteropancreatic neuroendocrine tumors (GEP-NETs) constitute a heterogeneous category of tumors originating from neuroectodermal cells. While the majority of GEP-NETs arise sporadically, some are associated with genetic syndromes such as neurofibromatosis type 1 (NF1), VHL syndrome, multiple endocrine neoplasia (MEN), and tuberous sclerosis (6). Previous case reports have indicated that VHL disease -lead to the development of pNETs (7). However, to the best of our knowledge, there is no empirical evidence directly associating VHL disease with gNET.

One case of gNET in the context of VHL was deemed to be associated with the utilization of proton pump inhibitors (PPIs). The follow-up after the discontinuation of the drugs revealed a reduction in lesion size until they became undetectable (8). Another gNET patient with VHL disease had chronic autoimmune gastritis, which was regarded as the more dominant carcinogenic factor (9). Contrary to these cases, our patient had no relevant drug history or other genetic background, thereby providing the first confirmation that gNET can independently serve as a rare clinical manifestation of VHL disease.

The pathological examination of the patient’s gastric lesion revealed hyaline change. This hyalinization is potentially attributed to HIF-α activation in VHL disease (10). Ordinarily, the VHL protein facilitates the degradation of HIF-α through an oxygen-dependent ubiquitin pathway. However, in VHL disease, mutations in the VHL gene compromise the inhibition of HIF-α, leading to its accumulation. This accumulation promotes the formation of HIF-α and HIF-β heterodimers, which activate the expression of various genes associated with tumorigenesis, including VEGF, EPO, TGF-α, and PDGFβ (11, 12). This mechanism also generates a pseudo-hypoxic environment, resulting in the accumulation of hepatic glycogen and fat, thereby causing cell hyalinization. This characteristic feature is observed in VHL-related tumors, such as pheochromocytomas and paragangliomas, especially in the context of multifocal pancreatic disease. Based on the pathogenic mechanism of HIF-α in VHL disease, HIF inhibitors were studied and evaluated in clinical trials. HIF-2α is a homolog of HIF-1α, and its hypoxia regulation is similar to HIF-α. Belzutifan, a second-generation oral HIF-2α inhibitor, was first approved in 2021 for the treatment of solid tumors in VHL patients. The latest extended follow-up results of LITIPARK-004 show that after a median follow-up of 49.9 months, the ORR was 91% in pNETs (13). Belzutifan exhibits sustained antitumor activity in the systemic treatment of VHL patients.

The pathological characteristics of NETs vary in accordance with their anatomical origin. Gastrointestinal NETs (GI-NETs) are typically confined to the submucosa or extend into the muscle layer, while pNETs commonly present as multinodular or infiltrative lesions (14). The majority of gNETs, except for the rare enterochromaffin or gastrin cell NETs, are composed of enterochromaffin-like (ECL) cells (15).

Furthermore, NETs that originated from the stomach and pancreas exhibit distinct microenvironmental and molecular characteristics. A study using single-cell RNA sequencing (scRNA-seq) highlighted the cellular transcriptome heterogeneity across anatomical subtypes of GEP-NETs (16). Concerning the tumor microenvironment (TME), T and NK cells are more abundant in GI-NETs, while myeloid cells predominate in pNETs. Disparities in the expression levels of specific mutant genes have also been observed. For instance, CHGA expression is higher in gNETs, whereas IGFBP2 is mainly expressed in pNETs. Subgroup analysis further revealed differences in lymphocyte subsets and the chemokine profiles of tumor-associated macrophages (TAMs) based on anatomical location. Additionally, DNA methylation patterns and SSTR subtype expression can serve as supplementary means to distinguish gNETs from pNETs (17, 18). This heterogeneity emphasizes that the clinical manifestations of gNETs in the context of VHL are distinctive and independent of those of pNETs.

Most cases of VHL syndrome are familial, with approximately 20% arising from sporadic mutations. The clinical manifestations exhibited by this patient are in line with typical VHL disease. The pathogenic mutation c.351G>T (p.Trp117Cys) has been documented in 12 cases within the UMD-VHL database. VHL disease patients typically possess heterozygous mutations in the VHL gene, signifying that the wild-type VHL gene remains functional even when the inherited germline mutation exists in all cell types (19). However, in accordance with Knudson’s “double hit” theory, tumor formation is triggered when a second mutation occurs in the wild-type allele, thus inactivating the tumor suppressor function of the VHL gene (20).

Once the patient was diagnosed with renal cancer, it became essential to manage the treatment for both GEP-NETs and renal cancer. Considering that the VHL mutation activates angiogenic pathways, antiangiogenic targeted therapies were regarded as appropriate for both tumor types. As a result, sunitinib was chosen for treatment. The patient achieved a progression-free survival of 26 months and an overall survival of 57 months after sunitinib treatment.

In this study, we performed genetic analysis on a patient exhibiting manifestations of VHL disease, verifying the existence of a pathogenic mutation in the VHL gene. We also inspected the morphological features of gNETs and highlighted their occurrence in patients with VHL disease. These findings suggest that VHL should be considered a potential differential diagnosis in patients with gNETs, particularly those who present with other VHL-related conditions.

## Conclusion

von Hippel–Lindau disease is an autosomal dominant disorder in which patients are at increased risk of developing ccRCC, central nervous system and retinal hemangioblastomas, as well as pheochromocytoma, paraganglioma, and pNETs. In this case, the patient underwent a gastroscopic biopsy after imaging examination showing tumor lesions in the stomach, liver, and kidneys. Pathology confirmed NETs and observed transparent changes. The 68Ga octreotide PET-CT, which is sensitive to NETs, confirms that the liver, rather than the kidneys, is the metastatic lesion. Based on the patient’s history of cerebellar hemangioblastoma, we considered that the patient is likely to have VHL disease, although there have been no reports of gNETs and VHL disease. Genetic analysis using peripheral blood DNA identified a germline mutation in the VHL gene. This timely diagnosis brings a longer treatment window for the patient. Genetic testing was also conducted on his family members, and the results suggest that they should closely monitor their health status and follow up regularly in the future. Given that gNETs are sporadic in VHL disease, we do not recommend gastroscopy as a routine screening modality for VHL patients. However, in Asian countries, considering the relatively high incidence of gastric cancer, gastroscopy serves a dual purpose; thus, it can be included as one of the surveillance items for VHL patients. The surveillance intervals should be individualized based on high-risk factors such as the patient’s age, for instance, every 1–3 years. Therefore, this case shows that gNETs could be considered as the atypical clinical manifestation of VHL disease for the first time. Clinicians should remain sensitive to similar cases to strive for early diagnosis and treatment opportunities.
